# Promising Strategies for Preserving Adult Endothelium Health and Reversing Its Dysfunction: From Liquid Biopsy to New Omics Technologies and Noninvasive Circulating Biomarkers

**DOI:** 10.3390/ijms23147548

**Published:** 2022-07-07

**Authors:** Carmela Rita Balistreri

**Affiliations:** Cellular and Molecular Laboratory, Department of Biomedicine, Neuroscience and Advanced Diagnostics (Bi.N.D.), University of Palermo, 90134 Palermo, Italy; carmelarita.balistreri@unipa.it

**Keywords:** endothelium, endothelium dysfunction, newer omics technologies, network medicine, biomarkers and therapeutic targets

## Abstract

The endothelium has multiple functions, ranging from maintaining vascular homeostasis and providing nutrition and oxygen to tissues to evocating inflammation under adverse conditions and determining endothelial barrier disruption, resulting in dysfunction. Endothelial dysfunction represents a common condition associated with the pathogenesis of all diseases of the cardiovascular system, as well as of diseases of all of the other systems of the human body, including sepsis, acute respiratory distress syndrome, and COVID-19 respiratory distress. Such evidence is leading to the identification of potential biomarkers and therapeutic targets for preserving, reverting, or restoring endothelium integrity and functionality by promptly treating its dysfunction. Here, some strategies for achieving these goals are explored, despite the diverse challenges that exist, necessitating significant bench work associated with an increased number of clinical studies.

## 1. Introduction

**Figure 1:**
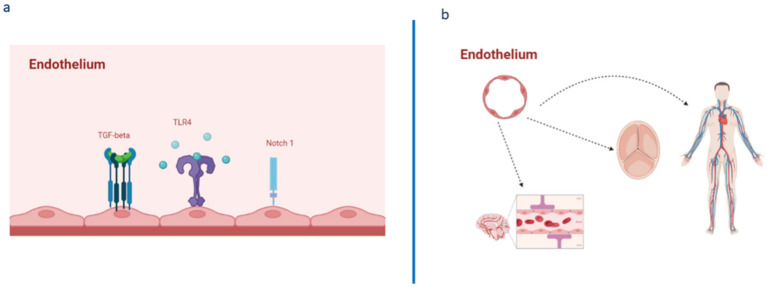
Architecture, EC pathways, and their heterogeneous presence and complexity in various body tissues. (**a**) Endothelium architecture and expression of some crucial pathways involved in its homeostasis, as well as in its activation, determining changes in the expression of specific genes and in the consequent phenotypic EC changes related to the evocation of dysfunction, caused by inflammation and endothelial barrier disruption (increased vascular permeability, edema formation, release of proinflammatory cytokines, and leukocyte extravasation). (**b**) The endothelium as an essential element of not only of the entire cardiovascular system, but also as the “heart” of all the tissues of the body, consequently contributing to their physiological homeostasis and function.

The term “endothelium” indicates the cellular layer that acts as barrier between blood flow and the inner lining of the vessel wall. Considered for a number of years as an inert vascular component, the endothelium is recognized today as the hub of the homeostasis of the entire cardiovascular system, including the heart valves, where endothelial cells (EC), defined as valvular ECs (VECs), can control the proliferation, differentiation, apoptosis, and remodeling of valvular interstitial cells (VICs) [1]. Thus, the endothelium represents a vital organ in the cardiovascular system and the principal element of the stroma of all body tissues, executing diverse regulatory activities thanks to the production and release of several endothelial-derived vasoactive factors, acting via endocrine, paracrine, or autocrine signaling. Accordingly, endothelial control includes not only the regulation of vascular tone, but also angiogenesis, inflammation, platelet aggregation, leukocyte adhesion, vascular cell proliferation, and oxidative stress [2,3,4,5,6] (see Figure 1). Such essential functions of the endothelium are already marked during fetal life. Such essential functions of the endothelium are already marked during fetal life. In the fetus, the endothelium has the fundamental role of ensuring the correct ontogenesis of immune cells (i.e., tissue-resident macrophages, microglia cells, and epidermal γδ T cells) and cells of the hematopoietic system, as well as the development of other systems, such as the nervous and cardiovascular systems [6,7,8].

**Figure 2:**
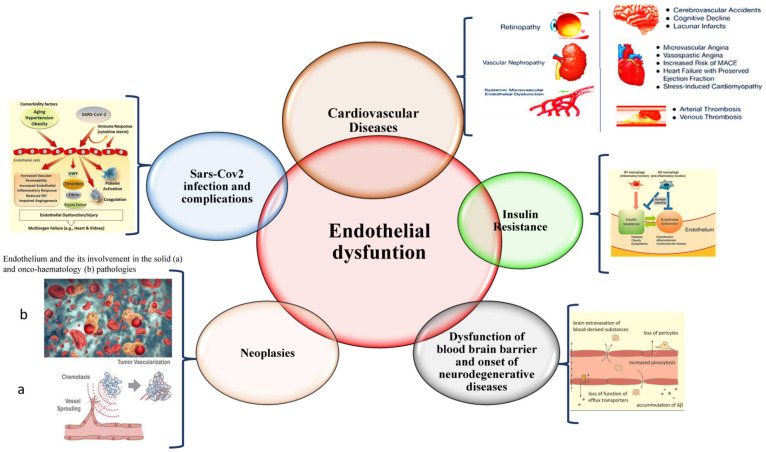
Endothelium dysfunction and its relationship with human diseases.

Consistent with these observations, the current evidence reports a close relationship between biochemical or structural injury, altered genesis, homeostasis, and function of the endothelium, and the onset and progression of pathological conditions correlated to inflammation, thrombosis, and atherosclerosis, as well as alterations in the vascular resistance and blood pressure [2,4,9,10,11]. Consequently, endothelial dysfunction is assumed as the axis of activation of all pathophysiological mechanisms and related disease pathways linked to excessive morbidity and mortality, such as hypertension, myocardial infarction, aneurysm, stroke, diabetes, metabolic syndrome, rheumatic diseases, renal failure, neurodegenerative diseases, cancer, and inflammatory diseases [2,4,5,8,9,11,12,13,14,15,16,17,18,19,20] (see Figure 2). For instance, tumor neovascularization is characterized by a highly disordered vascular endothelium network and the generation of new blood vessels. In addition, the abovementioned pathologies are characterized as coexisting in patients with a median age of about 65 years old and over by increasing the risk of adverse events [21,22]. Furthermore, a significant association between typical EC plasma proteins and individual risk of cardiovascular diseases (CVD), detected using the Framingham risk score, has also been evidenced in recent studies confirming the close relationship between CVD onset and endothelium dysfunction, as well as corresponding biomarker profiles [23]. Interestingly, alterations in the vascular endothelium have also been associated with organ dysfunction in patients affected by severe SARS-CoV-2 infection, belonging to the 70–89 age group [24,25,26,27] (see Figure 2).

Endothelial dysfunction constitutes a common pathological condition, which accompanies the aging process of all the tissues, organs, and systems, and the development of cardiovascular diseases including atherosclerosis, thrombosis, hypertension, aneurysms, valve diseases, but also of cancer, onco-haematology diseases, diabetes, and neurodegenerative disorders, as well as SARS-CoV-2 infection and related complications.

**Figure 3:**
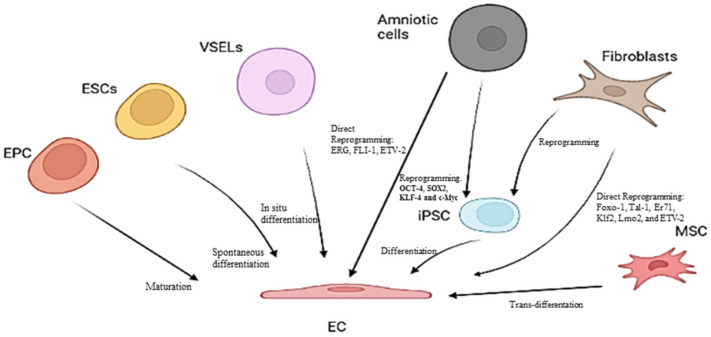
Some strategies for obtaining mature EC.

Considering these observations, it is of crucial importance to develop strategies with the aim of not only warding off aggressive agents or risk factors for preventing endothelium dysfunction but also identifying therapies which can reverse or mitigate the damaging effects generated by such conditions. Certainly, regenerative strategies via induced pluripotent stem cells (IPSCs) and direct reprogramming targeting ECs via transcription factors (TFs) involved in endothelial cell fate are optimal examples. TFs, such as OCT4, SOX2, KLF4, c-MYC, ETV2, and ERG, have recently become the subject of many studies, and the direct reprogramming strategy appears to be promising for the treatment of diseases such as CVD. However, limitations in their efficiency linked principally to epigenetic resistance and the methods used have been evidenced [28] (see Figure 3). Thus, the clinical applicability and translational nature of such strategies do not appear be evident. Here, attention is focused on new promising methodologies and biological matrices for use in achieving the suggested objectives. Accordingly, the use of blood liquid biopsy and omics technologies is described in the next section, in addition to their application in studies on endothelial dysfunction aimed at identifying biomarkers and emerging therapeutic targets.

Given the complexity of the mechanisms and related pathways related to the responses of the endothelium to physical or biochemical stimuli in carrying out its roles, diverse biomarkers and therapeutic targets are described and discussed with respect to their likely prevention and treatment of endothelium dysfunction and its pathological complications, while advantages and limitations are also reported.

## 2. Omics Technologies and Liquid Biopsy as Emerging Tools for Identifying Endothelial Biomarkers and Therapeutic Targets

**Figure 4:**
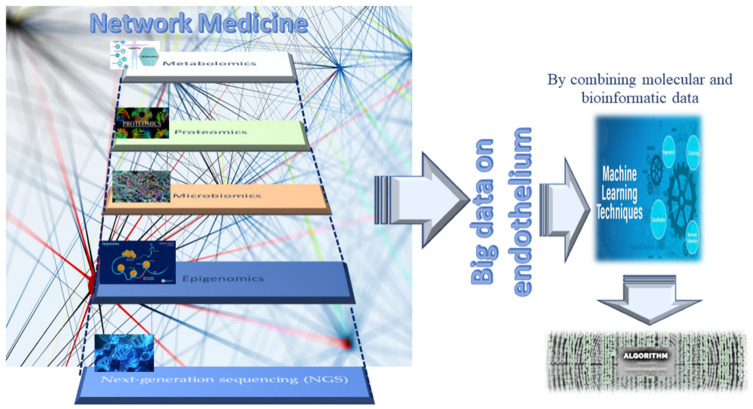
Network medicine and its features in endothelium studies.

In proposing new strategies for preserving the functionality and efficiency of adult endothelium tissue, it is imperative to identify molecules as optimal biomarkers and therapeutic targets. Biomarkers represent crucial indicators of both physiological and pathological processes, such as endothelium dysfunction. Specific changes in molecular and cellular mechanisms of physiological processes result in biochemical alterations at both the tissue and the systemic level, which can give us comprehensive information regarding the health status of tissues (in our case, the endothelium), as well as the nature of any pathological tissue dysfunction and disease. In addition, any disease biomarker should be specific and reliable, able to distinguish between the physiological condition of a tissue, organ, or system and a dysfunctional condition or disease, and diverse across various diseases or their subgroups and phenotypes. Accordingly, biomarkers can predict the risk of disease, facilitate early diagnosis, and enable setting guidelines for the development of new therapies for treating diseases. New-generation omics technologies and their platforms, including next-generation sequencing (NGS), epigenomics, proteomics, metabolomics, and microbiomics [29,30] (see Figure 4), can detect molecules involved in the close relationship between genotype and the environment, nutritional habits, and endothelial health or dysfunction, at an unprecedented grade of importance and resolution [31,32,33]. They also allow for obtaining an enormous quantity of data, albeit accompanied by consequent computational difficulties in their interpretation [31,32,33]. Today, such a challenge has been resolved thanks to potent bioinformatics algorithms, which can easily interpret and combine big data, by offering the possibility of both analyzing them at diverse multilevel networks and identifying genes, proteins, metabolites, and microbiomes, as well as their interactions in the complex human interactome, which can be used as emerging biomarkers and drug targets [31,32,33] (see Figure 4). Such a strategy exemplifies network medicine with the feature of combining molecular and bioinformatic data, which can improve current medical practices in the management of endothelial dysfunction-related pathologies by overcoming the limits of the obsolete points of view regarding endothelial dysfunction, considered the direct consequence of a single molecular alteration. An example of such an approach is machine learning (see Figure 4). The Abasht group [34] used this approach to evaluate vascular endothelial dysfunction in wooden breast syndrome, while the Treder group [35] used this approach to detect graft detachment in Descemet membrane endothelial keratoplasty. Furthermore, it is currently being applied to develop algorithms for vascular endothelial senescence, as recently evidenced by Sun and Feinberg [36].

Furthermore, omics technologies enable evaluating changes at a single-cell level, i.e., the evaluation of gene expression profiles of the endothelium at the transcriptome and epigenome level, when exposed to one or more stressors, such as disturbed blood flow, high-glucose status, or inflammation. Specifically, it is possible to evaluate the transcriptomic and chromatin accessibility variations at the resolution of a single-EC. This allows comparing the effects of stressors in healthy ECs vs. stressed ECs in the same vessel or in diverse vessels, thereby following the changes in physiological status of the endothelium as a result of dysfunction or other conditions, e.g., hyperplasia (see below). A similar study design was recently adopted by Kumar and coworkers [37] to evaluate the gene expression profile in ECs isolated, via an innovative protocol, from the lumen of carotid arteries from mice subjected to a partial carotid ligation (PCL), which induces disturbed blood flow (d-flow) in the left carotid artery, while maintaining stable laminar flow in the right carotid artery, used as a control. Such a mouse PCL model allows comparing the effects induced in ECs by blood flow under disturbed conditions or with stable laminar flow (s-flow). ECs were obtained from the carotid arteries by dissection 2 days or 2 weeks post PCL surgery, before subjecting their lumen to collagenase digestion to acquire endothelial-enriched single cells or single nuclei. These single-cell and single-nuclei preparations were utilized for RNA preparation, library generation, single-cell RNA sequencing (scRNA-seq), and single-cell transposase-accessible chromatin sequencing (scATACseq), which were used to determine the transcriptomic and chromatin accessibility changes. The data obtained consequently allowed evaluating the genes expressed under the effects induced by d-flow or s-flow at a single-cell level, resulting in the activation of proatherogenic or physiological responses. These proatherogenic genes and their molecules were then suggested as targets for developing therapies. Specifically, it was confirmed that Krüppel-like factors 4 and 2 (KLF4/KLF2) act as s-flow-sensitive transcription factor (TF)-binding sites. The identified TFs sensitive to d-flow were RELA, AP1, STAT1, and TEAD1, which were suggested to induce d-flow EC reprogramming from an athero-protective to a proatherogenic phenotype, including endothelial–mesenchymal transition [37,38]. Furthermore, thanks to the integration of diverse omics assays, including the acetylation of histone 3 lysine 27 (H3K27ac) ChIP-seq (chromatin immunoprecipitation followed by high-throughput sequencing), ATAC-seq (an assay for transposase-accessible chromatin sequencing), and RNA-seq (RNA-sequencing), it was possible to detect the genome-wide epigenetic regulations in ECs in response to athero-protective pulsatile shear stress (PS) [39]. The combined data obtained showed that inositol 1,4,5-trisphosphate receptor 3 (ITPR3) was upregulated under PS conditions via KLF4 or statins, in ECs isolated from mouse aorta, lung ECs isolated from EC-KLF4-transgenic versus EC-KLF4-knoukout mice, and atorvastatin-treated ECs. The use of KLF4 ATAC-qPCR (quantitative polymerase chain reaction) and ChIP-qPCR also led to the identification of a specific locus in the promoter region of the ITPR3 gene indispensable for KLF4 binding, H3K27ac enrichment, chromatin accessibility, RNA polymerase II recruitment, and ITPR3 transcriptional activation [39]. By evocating the deletion of KLF4 binding locus in EC thanks to the use of a clustered regularly interspaced short palindromic repeats-associated protein 9 (CRISPR/Cas9) system, a blunted calcium influx, reduced expression of endothelial nitric oxide synthase, and diminished nitric oxide bioavailability were evidenced. In the complex, such interesting results obtained thanks to novel multi-omics investigations suggested that KLF4 is crucial for PS-modulated H3K27ac, which allows the transcriptional activation of ITPR3. Such a mechanism contributes to Ca^2+^-dependent endothelial nitric oxide synthase (eNOS) activation and EC homeostasis, and these molecules are potential candidates for biomarkers and therapeutic targets [39].

Another group integrated investigations of in vivo epigenomic mapping with conditional knockout, gene transfer, and pharmacology with the aim of studying, in rodent models, the endothelial hyperplasia/proliferation (IH), which represents the primary etiology of vascular stenosis. The data from injured (IH-prone) rat arteries demonstrated a rise in genome-wide occupancy by histone 3 lysine 27 trimethylation (H3K27me3), a gene-repression indicator. This result was unexpected when considering the traditional point of view on prevailing post-injury gene activation rather than repression. Additional evaluation has also demonstrated a shift in H3K27me3 enrichment to anti-proliferative genes from pro-proliferative genes, characterized by gene activation via H3K27ac (acetylation) accumulation. H3K27ac and its reader BRD4 (bromodomain protein) showed co-enrichment of *Ezh2*, while conditional BRD4 knockout in injured mouse arteries resulted in a decrease in H3K27me3 and its writer EZH2, which positively regulates another pro-IH chromatin modulator, UHRF1. Thus, these results reveal an injury-induced locus-specific H3K27me3 redistribution in the epigenomic landscape involving BRD4 → EZH2 → UHRF1 hierarchical regulation, while suggesting that these players may be pharmaceutical targets [40].

Another interesting study was conducted in 2021 by a group in Napoli examining the glucose-dependent and dose-responsive alterations in endothelial DNA methylation to detect a putative epigenetic mechanism underlying diabetic vasculopathy. Specifically, they discovered the disproportionate glucose-dependent methylation and gene expression of VEGF and NO signaling cascades, constituting a physiological imbalance known to evocate endothelial dysfunction in diabetes. Consequently, they assumed that epigenetic mechanisms encode glycemic memory within EC, and the pathways involved can be used as therapeutic targets [41]. Specifically, their novel evidence demonstrated that hyperglycemia triggers dose-responsive variations in DNA methylation dynamics, affecting key physiological processes involved in the maintenance of endothelial function, including a glucose-dependent physiologic uncoupling of VEGF and NO signaling, which causes endothelial dysfunction.

Additionally, recent advances in molecular genetics and omics technologies also showed significant molecular heterogeneity within brain endothelial and perivascular cell types [42]. The blend of these conventional and modern approaches has enabled detecting phenotypical variations between healthy and abnormal conditions at the single-cell level. Accordingly, a deep understanding of brain vascular cell states during physiological, pathological, and aging processes has rapidly emerged, and therapeutic approaches have been developed, including vascularization models of human brain organoids on a chip (widely quoted in [42]), self-assembling multicellular blood–brain barrier (BBB) spheroids (widely quoted in [42]), and improved endothelial BBB differentiation protocols using human pluripotent or induced pluripotent stem cells (widely quoted in [8,42]). Human organoid and cell reprogramming technologies have rapidly improved, allowing the simulation of human brain development and disease modeling with the construction of a functional vasculature. These in vitro models will become potent instruments for exploring human-specific vascular traits absent in animal models, for investigating drug delivery across the BBB, and for screening drugs targeting BBB dysfunction in neurological diseases. Consequently, this research recently led our group, along with others, to hypothesize a close relationship between endothelial BBB conditions and neurodegenerative diseases (widely quoted in [8,42]).

**Figure 5:**
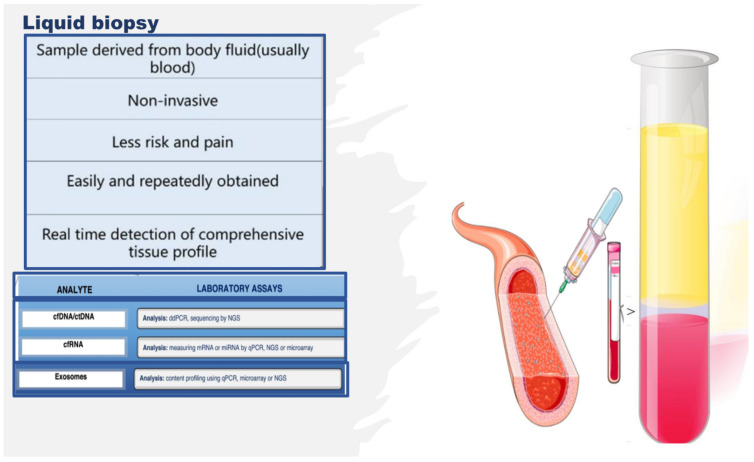
Liquid biopsy, describing some components and laboratory analysis techniques. Liquid biopsy involves the collection and analysis of diverse components from peripheral blood samples, including cell-free nucleic acids (cfDNA/ctDNA and cfRNA) and exosomes. cfDNA: circulating free DNA, ctDNA: circulating tumor DNA, cfRNA: cell-free RNA, CTCs: circulating tumor cells, TEPs: tumor-educated platelets, NGS: next-generation sequencing, qPCR: quantitative polymerase chain reaction.

Therefore, the literature is seeing an increase in the number of multi-omics investigations on endothelium, and the studies reported above constitute only some examples. However, many of these studies were complex with regard to their design, often invasive or necessitating the sacrifice of animals, along with an intricate approach to collecting the biological study samples and the related costs. Alternatively, studies on other types of samples, whose collection attracts a minor cost and a simple technique, are growing in number. Indeed, studies aimed at identifying noninvasive indicators of endothelial tissue status, simply using liquid biopsy, are increasing. Liquid biopsy is attractive as it facilitates the collection of an optimal biological sample, thereby enabling the extrapolation of big data via multi-omics technologies [43,44,45]. Liquid biopsy consists of an easy sampling of diverse bodily fluids, principally including blood, saliva, and urine, for the evaluation of various potential circulating noninvasive biomarkers [43,44,45]. As a biopsy, it also enables obtaining circulating intact cells, cell-free nucleic acids, circulating epigenetic-sensitive molecules (methylated DNA, modified histones, and noncoding RNAs), circulating metabolites, and other cell products, such as micro-vesicles and exosomes [43,44,45] (see Figure 5). However, unlike tissue biopsy, liquid biopsy offers the advantage of colleting heterogeneous cellular phenotypes by giving information on many circulating cell types and their products at specific times, thereby allowing the real-time monitoring of the evolution of a pathological condition or of the switching of the endothelium from physiological to dysfunctional [46]. In addition, emerging NGS platforms executed on body fluid samples, principally blood, can detect novel circulating noninvasive biomarkers, which may further the development of precision medicine and personalized therapy in the field of endothelium dysfunction-related pathologies, by preserving the health of the endothelium or reverting its alterations [43,44,45,46]. Furthermore, liquid biopsy shows several benefits compared to tissue biopsy; it is (a) noninvasive with negligible pain and risk, (b) more quickly executed, (c) easier to collect, (d) able to provide a real-time systemic profile, (e) able to give spatiotemporal information, and (f) able to monitor physiological or pathological conditions over time.

As mentioned above, fluid-based assays may provide noninvasive indicators that indicate the endothelium status or allow sequential monitoring of the evolution of dysfunction. Interesting evidence can be gained by assessing the number, function, and senescence grade of endothelial progenitor cells (EPCs), as well as by evaluating the effects induced by stem-cell therapies (i.e., human umbilical cord-derived mesenchymal stem cells (MSCs)) or by detecting the quantity and quality of circulating metabolites and other cell products, such as micro-vesicles and exosomes or molecules able to modulate the gene expression at transcriptional, post-transcriptional, and translational levels, including circular RNAs (circRNAs) and ADAR enzymes. The next section reports the description of some of these metabolites and cell products, stressing their emerging potential as biomarkers and therapeutic targets of endothelium dysfunction, as well as their related limitations.

## 3. Endothelial Progenitor Cells (EPCs) as Potential Biomarkers of Endothelium Dysfunction and Therapeutic Agents

Circulating EPCs represent the progenitors of ECs, and they originate from bone marrow (BM)-derived hematopoietic stem cells (HSCs) [47,48,49]. EPCs constitute a real resource of ECs, thus maintaining vascular and tissue homeostasis and appropriate oxygen regulation and transport. Accordingly, EPCs represent a reservoir of circulating cells able to target injury sites, restore endothelium integrity, and enable physiological activities [47,48,49]. The impact of EPCs in the vascularization process has been proven in both animal models and humans. Such evidence has also led to the assumption that reductions in EPC circulating number and/or alterations in their functions related to different causes could impact endothelium function and architecture, as well as the onset and complications of endothelium dysfunction and, consequently, the survival of affected persons. Increased or decreased circulating EPC levels, as well as alterations in their function (for a detailed description, we invite the reader to consult our book, see reference [48]), have indeed been associated with vascular endothelium aging and diverse endothelium dysfunction pathologies, including coronary artery disease, stroke, diabetes, systemic sclerosis, autoimmune disorders, and aneurysms [50,51,52,53,54,55,56,57,58]. Our group, for instance, recently evidenced that subjects affected by bicuspid aorta valve syndrome show a significant decrease in both the tissue and circulating levels of the Notch pathway, as well as a decrease in blood EPC number, compared to subjects with a tricuspid physiological valve, whether in the presence or absence of aorta aneurysm (AAA) [51,59]. In addition, we also evidenced, using both blood and human tissue aorta samples, that the unique independent risk predictors for vascular aging are age and a reduced EPC number, as well as reduced EPC migratory activity and senescence, mediated by high SA-β-Gal activity and high levels of TP53, p21, p16, and inflammatory genes [59]. Furthermore, we reported in another study [60] that a decreased circulating number of EPCs, associated with increased RDW values, augmented blood levels of high-sensitivity C-reactive protein, and reduced mean values of both leukocyte telomere length and telomerase activity, can predict both vascular aging and ascending aorta aneurysm (AAA) onset and prognosis. Consequently, we affirm that these factors might be used as an ideal biomarker profile for vascular aging, as well as for the diagnosis and outcome of sporadic AAA.

Such evidence might be of clinical relevance, and possible new recommendations and preventive measures might be applied. Accordingly, Hill and colleagues [61] already showed that the number of circulating EPCs represents a better predictor of vascular reactivity than conventional cardiovascular risk factors. Furthermore, a significant correlation between in vitro EPC senescence and endothelium dysfunction pathologies risk was also detected in blood donors [59]. Consequently, EPCs might be considered as an optimal predictive biomarker, as well as a diagnostic and prognostic biomarker of endothelium dysfunction and related pathologies. Certainly, ulterior studies are needed. However, such evidence has also led to the use of EPCs as therapeutic agents in the autologous or heterologous treatment of several endothelium dysfunction diseases, as well as in clinical trials, despite contradictory results being obtained. In particular, a favorable improvement in left-ventricular (LV) function in a rat model of myocardial infarction (MI) after intravenous injection of ex vivo expanded human CD34^+^ cells was reported [47,48,49]. Another study examined the effect of catheter-based intramyocardial transplantation in a swine model of MI, providing encouraging outcomes in favoring the application of EPCs as a potential cell therapy in clinical trials [47,48,49]. Naruse and colleagues conducted a study to assess the therapeutic treatment of diabetic neuropathy using in vivo expanded human EPC and streptozotocin-induced diabetic nude rats [62]. They observed numerous micro-vessels at the site of EPC injection [63]. Another group evidenced an improvement in neurological functions in chronic cerebral ischemic rats injected with CD34^+^ HSC cells, including EPCs [63]. The ability of EPCs to expand in culture under in vitro conditions represents another barrier for their therapeutic use. Genetically modified and ex vivo expanded EPCs may become promising agents able to appropriately rescue the impaired neovascularization process under disease conditions. In a rhesus model, ex vivo CD34^+^ cell transfection with recombinant non-replicative herpes virus vector and subsequent cell transplantation resulted in the expression of vector genes in angiogenic areas of skin autografts of rhesus macaques. Since CD34^+^ cells possess a natural angiogenic tropism to the injured endothelium, they may serve as ideal candidates for the delivery of genes into areas of angiogenesis [64]. These results encouraged the execution of clinical trials for evaluating the potential of EPCs to enhance endothelial integrity and vascularization at ischemia sites in patients with CVDs. Three different strategies were mainly applied: (a) the *administration of granulocyte-colony stimulating factor (G-CSF)* for verifying the recruitment of the patient’s own bone morrow resident progenitors, with two preliminary studies demonstrating increased LV function [65]; (b) *the intracoronary infusion of BM progenitor cells in patients with MI*, which demonstrated positive effects on LV function in three smaller studies [47,48,49]. Subsequently, two prospective large trials assessed significant LV function after 4–6 months of administration of BM progenitor cells. Ten recent and large trials confirmed the success and safety of this procedure with a follow-up over 1.5 years (widely quoted in [47,48,49]). In addition, intramyocardial and intracoronary administration has recently been suggested as a suitable strategy for the treatment of patients with refractory angina (widely quoted in [47,48,49]); (c) a more invasive strategy involving *the direct injection of cells into target tissues* [59]. This treatment (specifically, *transepicardial or transendocardial injection of unfractioned BM cells*) has been performed in patients with diffuse coronary artery disease and intractable angina with no option of recanalization. Ventricular function and physical capacity were observed to increase, but the small sample size of these studies necessitates confirmation in larger studies (widely quoted in [47,48,49]).

Studies using *autologous cell therapy are also interesting*. Accordingly, Yamamoto’s group executed an intramuscular injection of autologous BM-derived mononuclear cells containing 1% CD34^+^ cells in patients with chronic limb ischemia [66]. They quantitatively assessed the expression of EPCs and endothelial markers (i.e., CD133 and VE-cadherin) before the experiment and after the injection. Before investigation, the transcription of these molecules was undetectable. Autologous injection determined an elevation of EPC marker transcription. Thus, they established that autologous BM cells may be used in the therapy of patients with arterial diseases. A replication of these results was obtained by Lenk and colleagues [67]. Erbs and colleagues also used this autologous treatment in patients who underwent recanalization of chronic coronary total occlusion [68]. Autologous treatment with EPCs, expanded for 4 days in endothelium growth medium, improves coronary endothelium function and wall motion abnormalities, and it has a beneficial effect on metabolism in the target area in patients with symptomatic coronary atherosclerosis.

Despite these promising data, the clinical application of EPCs in exogenous or autologous cell therapy remains unclear for different reasons. Accordingly, the validity of their results is influenced by different factors: (a) the insignificant number of patients in these studies; (b) their missing randomization; (c) participation of a limited number of centers; (d) the imprecise phenotypic features of EPC utilized in the treatments, (e) the different methods of administration applied, and (f) the security and viability of the treatments [47,48,49]. Teratoma formation, immunoreactivity, or arrhythmias constitute the major unfavorable effects which have been detected after such treatments. In addition, other limitations have not been considered, including the limited number of EPCs in circulation, which usually requires their cultured expansion in a sufficient number of subpopulations from peripheral blood. The in vitro quantification of progenitor cells to obtain a quantity sufficient to utilize in therapeutic treatment can be modulated by phenotypic changes during their differentiation in vitro with the risk of obtaining senescent cells, requiring artificial cell pre-activation or stimulation for this approach. In addition to such evidenced limitations, another aspect influencing the results of this study is the lack of standardized criteria and a consensus for defining, characterizing, and identifying EPCs with well-established surface biomarkers, protocols, and methodologies. Consequently, other investigations are imperative [47,48,49].

### Other Candidates for Therapeutic Agents of Endothelium Dysfunction

Promising evidence has recently been reported on the great potential of human umbilical cord-derived MSCs (hucMSCs) in repairing diabetic vascular endothelial damage, by applying assays of resazurin staining, MTT cell viability, wound healing, transwell migration, and Matrigel tube formation on human umbilical vein endothelial cells (HUVECs), and by evaluating how hucMSCs work through the assessment of RNA sequencing (RNA-seq) and molecular experiments [69]. Specifically, the use of a conditioned medium of hucMSCs (MSC-CM) revealed that hucMSCs improved the cell viability, wound healing, migration, and angiogenesis of HUVECs damaged by high glucose via paracrine signaling, and the altered gene expressions of IL-6, TNF-α, ICAM-1, VCAM-1, BAX, P16, P53, and ET-1 were significantly restored by MSC-CM. RNA-seq incorporated with real-time PCR and Western blot clarified that high glucose activates MAPK/ERK signaling in HUVECs, while MSC-CM reverses the abnormal phosphorylation of ERK and overexpression of MKNK2, ERBB3, MYC, and DUSP5 in the MAPK/ERK signaling pathway [69]. Certainly, other studies are needed.

## 4. CircRNAs and Their Potential as Biomarkers and Therapeutic Targets

CircRNAs are an endogenous group of noncoding RNA molecules [70,71,72], originating from pre-mRNAs and produced by back splicing [72]. They were first observed in plant viroids [73] and eukaryotic cells [74], and eventually discovered in almost all organisms across the eukaryotic tree of life [75]. Their expression appears to be tissue- [76] and cell type-specific and correlated with the specific stage of development or physiological and pathological conditions [75]. Accordingly, growing recent evidence demonstrates the diverse regulator roles and versatile cell actions of such molecules [71,75]. For example, circRNAs can act as miRNA decoys [77], RNA binding protein (RBP) sponges [78] and protein scaffolds [79], and they can also be translated into proteins [80,81]. In addition, it was also evidenced that circRNAs play roles in a large range of biological processes, including cell proliferation, apoptosis, and senescence, showing abnormal expression in diverse human diseases, such as cancers [82,83], neurodegenerative diseases, cardiovascular diseases [84] and immune diseases [85]. Such evidence has led to the consideration of circRNAs as promising biomarkers and therapeutic targets for human diseases [86], due to their stability, elevated levels, and tissue- and cell-specificity [86]. Moreover, they may be easily detected in body fluids, such as urine, saliva, cerebrospinal fluid, or blood. Specifically, they are collected using liquid biopsy, which as mentioned above represents a revolutionary device in the management of diseases, facilitating diagnosis, prognosis, and treatments [44,45]. Specifically, peripheral blood represents the major fluid used in liquid biopsy investigations, where circRNAs can be cell-free or in blood cells [45]. Cell-free circRNAs can be observed in the plasma, serum, or blood endogenous vesicles, resulting from their secretion into the blood from diverse tissues. In addition, as mentioned above, they are characterized by a large stability and abundance, facilitating their status as biomarkers of physiological processes or diseases.

Furthermore, recent studies have suggested the role of circRNas in preventing or protecting the endothelium from further injury via senescence, remodeling, and progression in atherosclerotic tissue. Below, we report important data regarding this aspect.

### 4.1. CircRNAs and the Endothelium: Recent Literature Evidence

A growing number of studies have demonstrated the close association between the expression of specific profiles of circRNAs and various physiological and pathological endothelium conditions. Here, we report some recent studies. For example, a study by Wu and coworkers [87] demonstrated that circGNAQ shows a significantly low expression in senescent endothelial cells. Accordingly, circGNAQ silencing was revealed to evocate cell senescence, confirmed by the increase in senescence-associated β-galactosidase activity, the decrease in cell proliferation, and the inhibition of angiogenesis. Mechanistic investigations demonstrated that circGNAQ works in the endothelium as an endogenous miR-146a-5p sponge, enhancing the expression of its target gene *PLK2* by decoying miR-146a-5p and, consequently, delaying the endothelial cell senescence. Through in vivo studies, Wu’s group also revealed a significant association between circGNAQ overexpression and the inhibition of both endothelium senescence and potential progression in atherosclerosis [87]. Consequently, such data suggest that circGNAQ has important functions in endothelial cells, particularly in controlling senescence and the pathogenesis of atherosclerosis [87]. This also led to evidence that the management of circGNAQ might represent a potential therapeutic approach for limiting the senescence of the endothelium and the progression of atherosclerosis. In agreement with these data, it was also observed that circANRIL, derived from antisense noncoding RNA in the INK4 locus (ANRIL), can interact with PES1 to alter exonuclease-mediated pre-rRNA processing and ribosome biogenesis. Consequently, circANRIL can provoke apoptosis and impede cell proliferation in vascular smooth muscle cells and macrophages, promoting antiatherogenic functions [88]. Given the stability of circRNAs, circANRIL might also be used as a potential therapeutic target for the treatment of atherosclerosis. Li and coworkers reported that the circRNAs ABCA1 and KHDRBS1 were significantly upregulated in atherosclerotic aortic vessels and H_2_O_2_-treated mouse aortic endothelial cells (MAECs). Consequently, they impacted atherosclerosis and vascular endothelial injury by targeting the miR-30d-3p/TP53RK and miR-140-3p/MKK6 axes, as well as their downstream signaling pathways [89]. Likewise, Wang and coworkers [90], using human umbilical vascular endothelial cells (HUVECs) treated with oxidized low-density lipoprotein (ox-LDL), observed the role of circ_0124644 in promoting ox-LDL-induced endothelial injury of HUVECs through the miR-149-5p/PAPP-A axis, emphasizing its diagnostic and therapeutic application in atherosclerosis. In contrast, Yu’s group [91] demonstrated in human oxidized low-density lipoprotein (ox-LDL)-induced brain microvascular endothelial cells that circ_0003423 improved ox-LDL-induced injury by regulating the miR-589-5p/TET2 axis. In HUVEC cells treated with high-glucose-levels, Zhou and coworkers [92] observed an overexpression of circ_0008360. Accordingly, circ_0008360 knockdown reduced the high glucose-induced vascular endothelial dysfunction by regulating miR-186-5p and cyclin D2, suggesting that circ_0008360 might act as a target for the treatment of vascular endothelial dysfunction [92].

Furthermore, Huang and coworkers [93] discovered that circ-RELL1 has a proinflammatory role in ox-LDL-induced HUVECs using high-throughput circRNA microarray assays. Accordingly, knockdown of circ-RELL1 decreased ICAM1 and VCAM1 expression in ox-LDL induced endothelium inflammation by directly targeting miR-6873-3p in the cytoplasm. This reduction in the miR-6873-3p determines decreases in MyD88 (myeloid differentiation primary response 88) protein expression and MyD88 mediated NF-κB activation. Furthermore, circ-RELL1 could abrogate the inhibition of inflammation response by acting on miR-6873-3p. Thus, circ-RELL1 modulates endothelium inflammation via the miR-6873-3p/MyD88/NF-κB axis in ox-LDL induced endothelial cells, evidencing its role as a potential therapeutic candidate for endothelium inflammation in atherosclerotic cardiovascular disease [93].

### 4.2. Limitations and Future Perspectives

The elevated stability, great quantity, and spatiotemporally specific expression of blood circRNAs suggest these molecules as perfect biomarkers for endothelium investigations using liquid biopsy. However, a biomarker with broad clinical applications must have proven analytical validity, clinical validity, and clinical utility [94]. Consequently, several questions need to be studied before peripheral blood circRNA biomarkers can be translated into clinical practice. First, a blood circRNA-based gene analysis should prove its analytical validity within clinically relevant conditions. Even if significant improvements have been made in the recent years [95], the methods for identifying profiles of circRNAs are far from ideal. Forthcoming studies need to test the analytical performance of various circRNA profiling methods in clinical blood samples, such as RNA-seq, circRNA microarray, reverse transcription quantitative PCR (RT-qPCR) and RTddPCR [95]. In estimating analytical sensitivity and specificity, reference standards that can be specifically applied in circRNA discovery and profiling are needed (Hardwick et al., 2017). Furthermore, the procedure to uncover and confirm blood circRNA biomarkers needs to be standardized, including blood collection and preservation, circRNA extraction, library construction, and computational analysis [95,96]. With the use of a standardized procedure, future studies need to estimate the technical robustness and reproducibility of the proposed biomarkers within and between laboratories. In addition, the blood circRNA biomarkers identified in current studies are only preliminary biomarker signatures for endothelium dysfunction [96]. Lastly, to examine their clinical validity, more clinical samples are necessary to prove their sensitivity and specificity in a larger sample size.

## 5. ADAR Protein Family as Biomarkers and Therapeutic Targets

Adenosine to inosine (A-to-I) RNA editing represents a post-transcriptional process that allows for selectively converting adenosines to inosines in double-stranded RNA (dsRNA) substrates [97]. Such a process is executed by a highly conserved group of enzymes, i.e., members of the adenosine deaminase acting on RNA (ADAR) family. Specifically, ADARs can alter the splicing and translation machineries, double-stranded RNA structures, and binding affinity between RNA and RNA-binding proteins. All ADAR components are characterized by a structure with a common domain having a variable number of amino-terminal dsRNA-binding domains (dsRBDs) and a carboxy-terminal catalytic deaminase domain. In humans, there are three ADAR enzymes defined as ADAR1, ADAR2, and ADAR3 [97]. ADAR1 and ADAR2 are expressed in a ubiquitous manner in the tissues and act on numerous RNA substrates. ADAR3 is expressed only in the brain and appears to be inactive, as assessed on various RNA substrates [98]. In addition, two isoforms of ADAR1 have been identified, defined as ADAR1p150 and ADAR1p110, with cytoplasmatic and nuclear localization, respectively. The cytoplasmatic location of ARAR1p150 is unusual, because ADAR proteins generally map in the nucleus, where their presence is dependent on functional dsRBDs. dsRNAs originating from ribosomal RNAs (rRNAs) and small nucleolar RNAs (snoRNAs) can guide their location [97,98]), which seems to be a regulatory mechanism for the editing of premRNAs through enzyme sequestration. However, additional ADAR roles are emerging, linked to a large spectrum of genes and proteins, such as diverse cell pathways regulated by ADAR family proteins and related to phenotypic changes in tissue cells. Accordingly, it is possible to underline that ADAR activity is an essential mechanism in mammals and altered editing has been associated with several human diseases, ranging from neurological and neurodegenerative diseases (schizophrenia, Alzheimer’s disease, depression, epilepsy, amyotrophic lateral sclerosis (ALS), and systemic lupus erythematosus (SLE)) to cancers and cardiovascular diseases [99,100]. Such evidence has led to numerous efforts to modify ADAR activity in vivo and correct eventual RNA editing dysfunction.

### 5.1. ADAR Enzymes in the Endothelium Literature

Among the multiple roles of ADAR enzymes, it was evidenced by Zhang’s group [101] that ADAR1 shows the capacity to regulate endothelial cytokine activation. Specifically, they used HUVECs as an in vitro model to examine the role of ADAR1 in interleukin (IL)-1β-stimulated endothelial activation. They observed that stimulation with IL-1β affected the expression of ADAR1 and the adherence molecules VCAM-1 and ICAM-1 in HUVECs. Specifically, ADAR1 overexpression reduced the expression of VCAM-1 and ICAM-1, while ADAR1 knockdown augmented the expression of ICAM-1 and VCAM-1, as well as the recruitment of THP-1 monocytes. Furthermore, using a luciferase reporter assay, it was proven that ADAR1 can act as a direct target of miR-143 in HUVECs, and that miR-143 overexpression can exacerbate this condition, while miR-143 knockdown can stop IL-1β-induced activation of HUVECs. Moreover, ADAR1 overexpression was observed to mediate the augmenting effect of miR-143 overexpression on IL-1β-induced HUVEC activation. Specifically, it was demonstrated that ADAR1 overexpression could reduce this effect, while ADAR1 knockdown could increase the PKR, IκBα, and NF-κB phosphorylation generated by IL-1β. In addition, the authors demonstrated that blocking NF-κB signaling with specific NF-κB inhibitor PDTC (pyrrolidine dithiocarbamate) could block the IL-1β-induced HUVEC activation augmented by ADAR1 knockdown. Thus, these data suggest that ADAR1 can be targeted by miR-143 in regulating IL-1β-induced HUVEC activation, and that the NF-κB pathway can act as the downstream mediator of ADAR1. In conclusion, miR-143 and ADAR1 may represent therapeutic targets for endothelium dysfunction [101].

Another European group [102] discovered that EC showed increased microRNA editing under ischemia in vivo in a murine hindlimb ischemia model and ex vivo in human veins, associated with a differential expression of ADAR1 and ADAR2. Likewise, the Stellos group demonstrated increased ADAR activity in EC, that can control inflammation in atherosclerotic cardiovascular disease [103]. In addition, ADAR1’s function was also found to be associated with editing on specific RNA substrates such as cathepsin S (CTSS) and miRNA487b in an atherosclerotic state [104,105].

### 5.2. Limitations and Future Perspectives

The re-coding and editing events mediated by ADAR enzymes can likely impact the functions and activities of relevant proteins, RNAs, and miRNAs. RNA editing can play a part in endothelium dysfunction as reported above. Such A-to-I changes are part of an epigenetic process that can potentially be regulated and corrected. A feasible approach might be the modulation of ADAR expression (by overexpression or silencing) at a single-cell level. Alternatively, specific editing sites with a key role in endothelial dysfunction and complications could be targets of therapeutic strategies aimed at preventing such conditions, by rescuing the correct editing version. In this regard, it is worth noting that the inhibition of RNA editing at a specific site is possible (Schirle et al., 2010) thanks to small molecules (such as antisense oligonucleotides or helix-threading peptides) that can abrogate the duplex structure required for editing [106]. A recent revolutionary study by Montiel-Gonzalez and coworkers [107] reported innovative therapeutic strategies possible thanks to ADAR enzymes. The increasing importance of ADARs in the context of endothelium dysfunction and complications provides newfound hope of inducing important changes in the landscape of future biomedicine.

## 6. Vascular Endothelial Glycocalyx and Its Components as Emerging Endothelial Biomarkers and Therapeutic Targets of Endothelium Dysfunction

ECs owe their physiological, anti-inflammatory, and anticoagulant properties to the complex carbohydrate-rich layer covering their luminal surface, named the glycocalyx [108,109]. Recent evidence has suggested that glycocalyx shedding affects the endothelium, causing dysfunction and inflammation, which emphasizes the value of glycocalyx maintenance for preventing disease induction and progression [108,109]. Accordingly, the endothelial glycocalyx (EG) realizes diverse functions, including mechanotransduction, maintenance of vascular integrity and vascular tone, support of the production of nitric oxide (NO), and anti-inflammatory and anticoagulant properties by interacting with plasma proteins and plasma cells [108,109,110]. In addition, it is composed of a scaffolding of proteoglycans (PGs), glycoproteins (GPs), and glycosaminoglycans (GAGs) associated with the underlying ECs. PGs are composed of a core protein and covalently attached GAG chains, represented by syndecans and glypicans. Syndecans, observed on the surface of most body cells, are transmembrane proteins with extracellular, transmembrane, and cytosolic domains [108,109,110]. The extracellular domain binds GAGs and detects extracellular signals, such as shear stress, which are transduced to the intracellular environment via the transmembrane portion and the cytoplasmic tail. There are four well-known syndecans in humans, syndecans 1, 2, 3, and 4; however, the EG primarily contains syndecan 1 (SDC1). Glypicans are not transmembrane proteins; they are instead attached to the EC luminal membrane via a glycosylphosphatidylinositol anchor. There are six well-known glypicans, with glypican-1 expressed on the endothelium [108,109,110].

Diverse factors, including disturbed or reduced flow profiles, vascular aging, diabetes, and obesity, determine biochemical and structural alterations in EG associated with the onset of endothelium dysfunction [108,109,110,111]. In addition, recent evidence has shown that EG is considered as one of pathological conditions commonly accompanying an ischemia/reperfusion injury (IRI), along with tissue damage due to ROS produced upon reperfusion, mitochondrial dysfunction with opening of the mitochondrial transition pore and loss of ATP production, activation of the complement system, and endothelial dysfunction [112,113]. Animal models of cardiac IRI have demonstrated an early reduction in the thickness of EG, precisely 5 min after reperfusion, whereby EG shedding causes a reduced vasodilation of the endothelium mediated by NO. In addition, it was demonstrated that the EG can regenerate over time; specifically, some in vivo studies have revealed that a period of about 7 days is sufficient to reestablish the EG layer. However, the mechanism underlying EG restoration. and whether its physiological composition is maintained, has not yet been explored [113]. However, various preventive and restorative approaches, with the aim of recreating physiological EG functions, have been proposed as potential therapies for endothelial dysfunction and its complications. Among these, the application of diverse molecules, i.e., angiopoietin-1 [114], hydrocortisone [115], ATIII [116], and SOD [117] increased the vascular integrity and prevented endothelial dysfunction during an IRI by inhibiting the degradation of glycocalyx components, namely syndecan-1, heparan sulfate and hyaluronic acid; therefore, they have been proposed as potential therapeutic candidates. However, how these molecules maintain EG integrity is still ambiguous.

Furthermore, many studies have demonstrated that some drugs [118], restoration with plasma [119], heparin [120,121], sulodexide [122], and hydroxyethyl starch can downregulate heparinase, hyaluronidase and neuraminidase [123], during inflammation or sepsis, consequently preventing endothelial dysfunction by preserving EG integrity. However, in this case, the corresponding mechanism of action also remains unclear. Thus, further studies are needed to confirm such a hypothesis.

## 7. Circulating Metabolites

Numerous metabolic dysregulations accompany the dysfunctional endothelium, as well as its related pathologies. Metabolites constitute both small molecules of the human metabolome, including amino acids, organic acids, nucleic acids, fatty acids, amines, sugars, vitamins, cofactors, pigments, and antibiotics (i.e., endogenous metabolites) and exogenous chemical metabolites (i.e., drugs, environmental contaminants, food additives, toxins, and other xenobiotics) produced by metabolic reactions. Thanks to diverse advanced metabolomics platforms, including nuclear magnetic resonance (NMR) spectroscopy, mass spectrometry (MS), liquid chromatography (LC), gas chromatography, and capillary electrophoresis, it is possible to both quantify and separate these compounds.

Some research groups have suggested the presence in circulation of diverse profiles of metabolites in the presence or absence of endothelial dysfunction or related pathologies. The Kots group [124] evaluated the associations of circulating levels of stable metabolites of nitric oxide, nitrate, nitrite (NOx), and endothelin-1, as well as the endothelin-1/NOx ratio, with blood pressure in 177 asymptomatic subjects without signs of coronary atherosclerosis, and in 457 patients with the presence of coronary lesions and suspected to have coronary heart disease with or without coronary lesions confirmed by coronary angiography. They observed that men had NOx levels inversely correlated with blood pressure, as did women with a low (0–4%) European Systematic Coronary Risk Estimation (SCORE). Furthermore, a significant negative association was evidenced between high systolic blood pressure over 140 mm Hg and NOx levels in asymptomatic men (*p* = 0.05), but not in women. In addition, in male patients, NOx (*p* = 0.05), endothelin (*p* = 0.01), and the endothelin/NOx ratio (*p* = 0.04) were significantly associated with coronary lesions.

Beckman and coworkers [125] recently demonstrated in patients affected by type 2 diabetes and healthy controls the presence of different metabolites in plasma samples, including homocysteine, dimethylguanidino valeric acid and β-alanine (all *p* < 0.05). Furthermore, they evidenced the presence of changes in the levels of ischemia-induced metabolites between the two groups including 5-hydroxyindoleacetic acid (healthy: −27%; DM +14%), orotic acid (healthy: +5%; DM −7%), trimethylamine-N-oxide (healthy: −51%; DM +0.2%), and glyoxylic acid (healthy: +19%; DM −6%) (all *p* < 0.05). Plasma amounts of serine, betaine, β-aminoisobutyric acid and anthranilic acid were significantly associated with vessel diameter at baseline, but only in T2DM cases (all *p* < 0.05). The results suggest that dysregulated muscle metabolism in T2DM may have direct effects on vascular function.

The Wang group [126] evidenced that a healthy endothelium releases metabolites able to modulate inflammatory responses and protect against systemic inflammation. Specifically, they detected, in conditioned endothelium extracts using liquid chromatography-mass spectrometry, the presence of 5-methoxytryptophan (5-MTP), but not other related tryptophan metabolites. Furthermore, it was observed that endothelial cell-derived 5-MTP can suppress lipopolysaccharide-induced inflammatory responses and signaling in macrophages and endotoxemic lung tissues. Thus, it was suggested that 5-MTP may be considered as part of a novel class of endothelium-derived protective metabolites that defend against endothelial barrier dysfunction and excessive systemic inflammatory responses.

Such results are promising; however, a larger number of studies are needed to confirm the role of such or other metabolites as biomarkers related to a healthy or dysfunctional endothelium. In addition, it is known that metabolomics deals with the end-products of gene expression in cells; consequently, this aspect needs to be strongly clarified, as well as the relationship among genetic variations, environmental factors, and metabolite endothelial profiles. Thus, further longitudinal studies may reveal novel metabolic pathways and biomarkers for improving personalized therapy of endothelium-related diseases.

## 8. Suggestions and Recommendations for Further Investigations Needed to Develop Biomarker Endothelial Profiles

The choice of and progress in identifying endothelial biomarkers should be based on a clear knowledge of the mechanisms and pathways related to the maintenance of endothelial health or its dysfunction. With that in mind, the several pathways and molecules described above have not completely achieved this purpose. They can clarify only part of the strong relationships between endothelium dysfunction and the onset of related diseases in various body tissues. For example, they do not allow for revealing the severity of injury, which would help practitioners to immediately intervene with personalized treatments or predict complications. In addition, given the long-term functional impairments of the endothelium throughout adult life caused by multiple factors and related inflammation, identifying diagnostic biomarkers to correlate with the severity of injury, prognostic biomarkers for the early prediction of related diseases, and diagnostic biomarkers for monitoring the progression of the dysfunctional endothelium, along with regression following appropriate treatments, could serve to strongly reduce the high incidence of frequent endothelial-related diseases in the elderly. Furthermore, understanding the dynamic and temporal biomarker levels might enable the development of a potent algorithm and reveal novel putative therapeutic targets.

However, these observations suggest that further scientific efforts are needed for the identification of biomarkers and targets for the development of therapies. To achieve this goal, further advances should be realized using a new technological appraisal based on innovative approaches and systems, as mentioned above. The integration of multi-omics analyses (genomics, epigenomics, transcriptomics, and proteomics, along with metabolomics, microbiomics, and nutrigenomics) and the introduction of new-generation multi-omics technologies are encouraged [30]. In the next section, we describe some of these promising technologies.

## 9. Innovative Technologies for Targeted Endothelium Treatments: Nanomedicine and Its Benefits and Limitations

**Figure 6:**
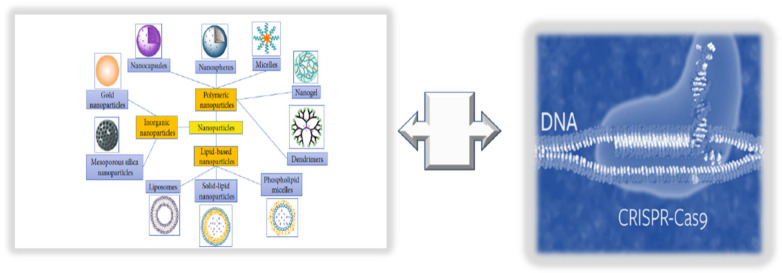
Innovative technologies for targeted endothelium treatments: nanomedicine + CRISPR/Cas9 editing.

The term nanomedicine indicates a specific targeted treatment, which can enhance the delivery of targeted drugs (i.e., natural antioxidant compounds or pharmacological drugs) and their bioavailability, as well as reduce the associated toxicity or side-effects and costs, using nanoparticles (NPs) [127]. NPs comprise liposomes, niosomes, polymers, lipid and organic polymer hybrids and precursors, carbon nanotubes, quantum dots, metals, and metal oxides (see Figure 6).

Thus, they constitute specialized carriers with the potential to facilitate the delivery of drugs and efficient molecular targets into desired tissues, such as the endothelium. Furthermore, NPs show another benefit in improving the effectiveness of therapeutic compounds, i.e., by decreasing their toxicity or side-effects. To this aim, NPs feature two aspects: (1) a versatile preparation method and a structure that allows encapsulating therapeutic agents, and (2) a functional surface able to interact with antibodies, aptamers, small molecules, etc., to further facilitate the delivery of therapeutics or diagnostics to pathological sites of the endothelium [127,128]. Accordingly, the linking of NP surfaces with molecular fractions mimicking natural ligands can provide selective targeting and precise delivery of therapeutics to a diseased tissue. Nevertheless, only a small percentage of these NPs can successfully accumulate in the selected tissues. For example, Wilhelm and coworkers [129] showed that fewer than 1% of nanoparticles manage to accomplish their role, specifically related to tumoral tissues. The reasons are diverse and mostly related to multiple physiological barriers and the high degree of stochasticity involved in NP activity. Accordingly, a high percentage of NPs are phagocytized by the mononuclear phagocytic system (MPS), while some remain physically “entrapped” in the sinusoids of the liver and others are taken up by hepatocytes and Kupffer cells [127]. In addition, NPs are incapable of adequately negotiating other biological barriers. This has hampered their clinical translation. Consequently, innovative biomimetic strategies [127] have been developed. They are mainly based on two approaches: (1) top-down approaches, including the bioengineering of pathogens (bacteria and viruses) or cells (leukocytes, erythrocytes, platelets, and stem cells) [130], or (2) bottom-up approaches, such as conjugating NP surfaces with analogs of bioactive molecules that bind CAMs and selectins [131,132] or coating synthetic particles with cell membranes, as reported by Parodi and coworkers [133]. Additional approaches include poly(lactic-co-glycolic acid) (PLGA) NPs coated with a platelet cell membrane that preferentially bind to the denuded artery in a rat model of coronary restenosis [131]. Other biomimetic NPs exhibit endogenous surface molecules, such as LDL and HDL, which have been used for incorporating nucleic material and delivering both therapeutic and diagnostic molecules [134].

Another important issue is the assessment of NPs’ biodistribution following in vivo administration in animals and humans [135,136]. This constitutes a great challenge despite the large range of techniques available for detecting nanoparticle biodistribution, including histology, electron microscopy, liquid scintillation counting (LSC), indirect measurement of drug concentrations, in vivo optical imaging, computed tomography (CT), magnetic resonance imaging (MRI), and nuclear medicine imaging.

However, a biomimetic innovative approach has been utilized to improve the bioavailability, stability, and consequent advantageous actions of curcumin on endothelial related diseases [136]. Similarly, pH and ROS dual-responsive NPs fabricated by incorporating pH- and ROS-responsive cyclodextrin materials with resveratrol were utilized as an efficient and secure nanoplatform for therapeutic delivery to areas of vascular inflammation in the presence of acidosis and oxidative stress [137,138]. Cho and co-workers also used biomimetic NPs with synthetic HDL (liposomal formulation with dimyristoyl phosphatidylcholine (DPMC)), demonstrating that they could significantly enhance the blood HDL levels, as well as reduce the plaque development in an animal study [139]. In another study, using a murine model of inflammation, the researchers targeted E-selectins by conjugating anti-E-selectin monoclonal antibodies to ultra-small-SPION in vivo for imaging of the endothelium to detect vascular inflammation [31]

### 9.1. Consideration of NPs as New Endothelium Treatment Strategy

Currently, newer treatment strategies, with reduced risks of evocating adverse effects, are being investigated, among which nanomedicine shows a more targeted specificity and efficacy. Thanks to the discovery of various endothelium-targeting molecules, efforts to design NP treatments targeting specific endothelium sites and ligands using appropriate nano-carriers are increasing. However, there are several challenges, including the shear stress under continuous flow, the variable size of arteries and their marginalization from the bloodstream, and the elimination of NPs by macrophages, which affect NP internalization in the endothelium. Consequently, numerous delivery strategies have been developed to improve the adequate transport of medication into specific endothelial regions, as mentioned above, including the production of biomimetic NPs. However, the number of translational studies is inadequate for elucidating the safety and efficacy of such NPs, and their delivery strategies remain a challenge. To progress to clinical application, NPs should interact with and internalize in the endothelium effectively, and they should be proven to be nontoxic, more successful than existing options, easy to use, and cost- and design-effective. Nevertheless, significant benchwork associated with an increased number of clinical studies may provide the necessary hope for treating endothelium dysfunction with nanotechnology [140].

Below, we evidence how the integration of NPs with other newer omics technologies can be used to successfully recovering endothelium function in adults.

### 9.2. Integrating NPs with CRISPR/Cas9 Editing and Base Primers

In the case of the endothelium, NP application shows several limitations, as largely stressed above. While developing such treatments, it is imperative to consider the high degree of endothelium heterogeneity and complexity, owing to its diverse morphology, location, and function shown in various body tissues. Endothelial alterations are directly or indirectly involved in a wide variety of human disorders, encompassing several mechanisms and pathways (including inflammation), and they are caused by and promote the progression of endothelial dysfunction. This can severely affect other cellular types, in physiological or pathological conditions, impacting the function of organs and systems, as well as physiological processes, such as regeneration, repair, cardiovascular genesis, neurogenesis, and cardiovascular and neural survival. This is significantly related to the altered gene expression of cells linked or not to genetic variants and/or genomic modifications. Consequently, it is hypothesized that a strong genetic editing technique might be a solution, as well as the integration of gene-editing techniques with nanotechnology. Accordingly, the clustered regularly interspaced short palindromic repeats-associated protein 9 (CRISPR/Cas9) system and techniques to chemically modify RNAs are also being incorporated in NPs to study human pathologies and/or examine potential disease correction through restoration [141,142]. The CRISPR/Cas9 system is significantly utilized for both editing the genome of zygotes, thereby generating genetically modified animal species, and treating human diseases. In fact, the development of safe and efficient treatments is becoming a reality by delivering the CRISPR/Cas9 system into body tissues and targeting specific cells [143,144]. A delivery system with optimal in vivo genome-editing efficacy is based on the use of the recombinant adeno-associated virus (AAV), but it can cause adverse effects. Thus, lipid NPs represent an alternative. For example, lipid NPs were used to target macrophages in mice using the CRISPR/Cas9 system, although a reduced genome-editing percentage (20%) was obtained. This suggests that it is a challenge to induce robust genome editing in the targeted endothelium. However, hope can be derived from a recent study conducted by the Zhang group on ECs obtained from the lung, heart, aorta, and peripheral vessels of adult mice. Specifically, Zhang and coworkers [145] developed poly(ethylene glycol) methyl ether-*block*-poly(lactide-*co*-glycolide) (PEG-*b*-PLGA; PP)-based NPs with excellent biodistribution for vascular delivery and demonstrated that the polyethyleneimine (PEI)-formulated PP NP-mediated delivery of the all-in-one CRISPR plasmid DNA expressing Cas9 under the control of the human CDH5 promoter and a guide RNA (gRNA) driven by the U6 promoter determined highly efficient genome editing in ECs of various vascular locations with a single administration. A reduction in protein expression of about 80% was evidenced in ECs. The potential demonstrated by such an approach could allow efficiently modulating or eliminating the expression of genes encoding proteins strongly associated with phenotypic changes related to the development of endothelium dysfunction and its related pathologies [145]. Today, several new CRISPR-based modalities have been developed that are capable of facilitating gene editing without the requirement of a DNA double-strand break (DSB), including base editors, prime editors, and RNA-targeting CRISPR-associated protein (Cas)13 effectors [146]. These modalities can potentially be integrated with NPs. However, this will require completely optimizing individual base editor proteins for specific therapeutic targets to guarantee maximally efficient formation of the on-target product, as well as minimize potentially counterproductive nontarget editing outcomes. In addition to developing methods to further refine the capabilities of these increasingly sophisticated gene-editing machines, special attention must be given to their delivery. Thus, further studies are needed.

## 10. Conclusions

**Figure 7:**
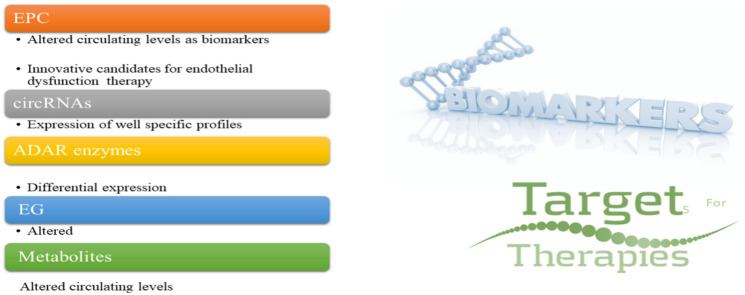
Innovative non-invasive biomarkers and therapeutic targets.

Individualized profiling of molecular patterns identified in bodily fluids represents a revolutionary approach in the framework of detecting biomarkers and developing therapeutic targeted treatments for preserving, reverting, and restoring endothelium barrier integrity and function. Currently, EPCs, circulating metabolites, EG components, circRNAs, and ADAR enzymes are attracting substantial attention for diagnostic and treatment purposes (see Figure 7).

Altered profiles of such molecules and cells have been detected under both physiological and pathological conditions of the endothelium. However, advanced implementation of such research is imperative for achieving these purposes independently of the application area.

This article highlighted the involvement of such molecules and cells in the healthy and diseased endothelium by addressing whether specific patterns of such molecules and cells in fluid biopsy and the use of newer omics technologies or their combinations can be further applied to accompany diagnosis, targeted prevention, creation of individualized therapy algorithms, therapeutic monitoring, and prognosis. The presented considerations correspond to the principles of network and personalized medicine, and they can be ameliorated to increase individual long-term survival outcomes, thereby guaranteeing a high quality of life and improved cost efficacy of medical services provided to the population.

## Data Availability

Not applicable.
